# High expression of MKP1/DUSP1 counteracts glioma stem cell activity and mediates HDAC inhibitor response

**DOI:** 10.1038/s41389-017-0003-9

**Published:** 2017-12-14

**Authors:** Olatz Arrizabalaga, Leire Moreno-Cugnon, Jaione Auzmendi-Iriarte, Paula Aldaz, Inmaculada Ibanez de Caceres, Laura Garros-Regulez, Veronica Moncho-Amor, Sergio Torres-Bayona, Olga Pernía, Laura Pintado-Berninches, Patricia Carrasco-Ramirez, María Cortes-Sempere, Rocío Rosas, Pilar Sanchez-Gomez, Irune Ruiz, Helena Caren, Steven Pollard, Idoia Garcia, Angel-Ayuso Sacido, Robin Lovell-Badge, Cristobal Belda-Iniesta, Nicolas Sampron, Rosario Perona, Ander Matheu

**Affiliations:** 1 Cellular oncology group, Biodonostia Health Research Institute, San Sebastian, Spain; 2 Centro de Investigación Biomédica en Red de fragilidad y envejecimiento saludable, Madrid, Spain; 3 Cancer Epigenetics Laboratory, INGEMM, IDIPAZ, Hospital La Paz, Madrid, Spain; 4 The Francis Crick Institute, London, UK; 5 Donostia Hospital, San Sebastian, Spain; 6 Instituto de Investigaciones Biomédicas CSIC/UAM, 28029 Madrid, Spain; 7 Neuro-Oncology Unit, Instituto de Salud Carlos III-UFIEC, Madrid, Spain; 8 Department of Pathology, Sahlgrenska Cancer Center, Institute of Biomedicine, Sahlgrenska Academy, University of Gothenburg, Gothenburg, Sweden; 9 MRC Centre for Regenerative Medicine, Edinburgh, UK; 10 IKERBASQUE, Basque Foundation for Science, Bilbao, Spain; 11 Centro Integral Oncológico Clara Campal (CIOCC) and Instituto de Medicina Molecular Aplicada (IMMA), Madrid, Spain; 12 Centro de Investigación Biomédica en Red de Enfermedades Raras, Madrid, Spain

## Abstract

The elucidation of mechanisms involved in resistance to therapies is essential to improve the survival of patients with malignant gliomas. A major feature possessed by glioma cells that may aid their ability to survive therapy and reconstitute tumors is the capacity for self-renewal. We show here that glioma stem cells (GSCs) express low levels of MKP1, a dual-specificity phosphatase, which acts as a negative inhibitor of JNK, ERK1/2, and p38 MAPK, while induction of high levels of MKP1 expression are associated with differentiation of GSC. Notably, we find that high levels of MKP1 correlate with a subset of glioblastoma patients with better prognosis and overall increased survival. Gain of expression studies demonstrated that elevated MKP1 impairs self-renewal and induces differentiation of GSCs while reducing tumorigenesis in vivo. Moreover, we identified that MKP1 is epigenetically regulated and that it mediates the anti-tumor activity of histone deacetylase inhibitors (HDACIs) alone or in combination with temozolomide. In summary, this study identifies MKP1 as a key modulator of the interplay between GSC self-renewal and differentiation and provides evidence that the activation of MKP1, through epigenetic regulation, might be a novel therapeutic strategy to overcome therapy resistance in glioblastoma.

## Introduction

Glioblastoma is the most common and malignant brain cancer in adults, which is characterized by its intrinsic aggressiveness and dismal prognosis^1^. Current therapy consisting of surgery followed by radiotherapy and chemotherapy with temozolomide has partial effectiveness, but the tumor recurs. Thus, overall patient survival is 15 months and percentage of survivors at 3 years is around 5%^2^. Glioblastoma is characterized by significant heterogeneity at clinical, morphological, molecular genetic, and cellular levels, and this heterogeneity is a major explanation for the poor prognosis. In particular, it has been demonstrated that treatment failure is due to the inability of current therapies to eliminate a subpopulation of glioma cells, with stem cell characteristics called glioma stem cells (GSCs)^3^. GSCs contain self-renewal capacity and can give rise to the various cell lineages that comprise the tumor^4–7^; therefore, they have been postulated as responsible for the origin, maintenance recurrence, and drug resistance of glioblastoma^8^.

Mitogen-activated protein kinases (MAPKs) are protein kinases involved in intracellular signaling during proliferation, differentiation apoptosis, and cell stress responses. Activation of MAPKs, involving ERK1/2, p38 MAPK, JNK, has been implicated in the development and progression of several cancers, including glioblastoma^9–11^, and for resistance to chemotherapeutic DNA-methylating agents^12, 13^. Recently, it has been found that the activation of MAPKs is necessary for GSCs self-renewal activity, the ability to initiate tumors and radioresistance^14–17^, leading to the evidence that GSCs maintenance via MAPKs is a major step in the progression and therapy resistance of glioblastoma.

MAP kinases are regulated by the family of MAPK phosphatases (MKPs), also called dual-specificity phosphatases (DUSPs), which are able to dephosphorylate both the threonine and tyrosine residues in conserved motifs, thereby inhibiting their activity^18^. Among them, MKP1/DUSP1 is expressed ubiquitously and its transcription increases rapidly in response to growth factors, oxidative stress, heat, hormones, cytokines, osmotic stress, hypoxia, and chemical and physical DNA damage. MKP1 has nuclear localization and its major substrates are JNK, p38 MAPK, and ERK1/2. The preference for each of these substrates is dependent on the tissue or also specific cell types. MKP1 plays an important role in oncogenesis, tumor progression, and resistance to chemotherapy in cancers such as ovarian, lung, or breast^19–21^. However, its function in cancer stem cells has not been identified and its role in glioblastoma remains unknown.

## Results

### High levels of MKP1 correlate with extended glioblastoma patient survival

**Fig. 1:**
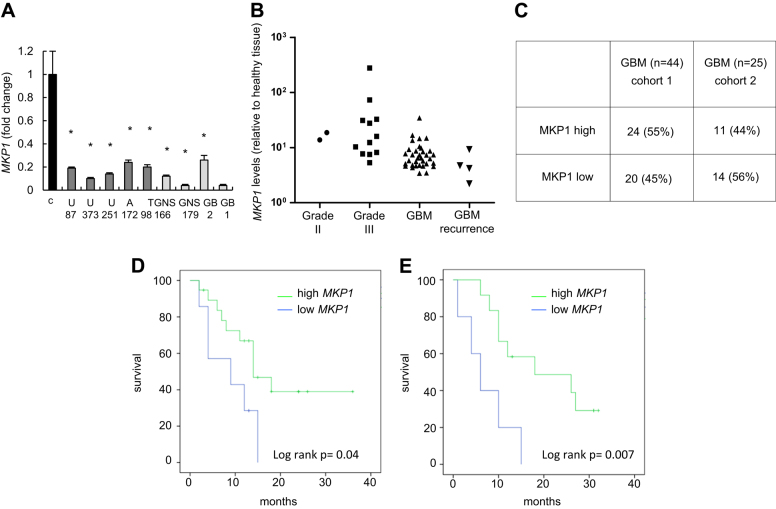
**High levels of MKP1 correlate with extended glioblastoma patient survival. a** *MKP1* mRNA levels were assayed in a set of healthy brain tissues as control (*n* = 6), glioma cell lines (U87MG, U373MG, U251, A172, and T98), and patient-derived GSC cell lines (GNS166, GNS179, GB2, and GB1). qRT-PCR data are normalized to *GAPDH* expression. **b** *MKP1* mRNA levels were assayed in a cohort including a set of healthy brain tissue as control (*n* = 6) and glioma samples (*n* = 59, 44 of them were GBM). qRT-PCR data are normalized to *GAPDH* expression and expression in tumors is relative to healthy brain tissue. **c** Analysis of the correlation of MKP1 expression with different glioma grade samples in two independent cohorts. **d** Kaplan–Meier curve representing the survival of the GBM patients relative to their MKP1 expression levels (*n* = 44). **e** Kaplan–Meier curve representing the survival of the GBM patients relative to their MKP1 expression levels (*n* = 25)

We first analyzed *MKP1 expression* levels by RT-PCR in a set of glioma cell lines and found that *MKP1* expression was generally low on glioma cells compared to normal brain tissue (Fig. 1a). Next, we moved to patient-derived GSCs observing that four different cultures, two grown as adherent lines (GNS166, GNS179) and another two as oncospheres (GB2 and GB1) expressed even lower *MKP1* messenger RNA (mRNA) levels than conventional glioma cell lines and healthy brain tissue (Fig. 1a).

We also studied *MKP1* expression levels in a set of glioma human samples containing grade II–IV biopsies and compared them to non-neoplastic brain tissue from Valencia Hospital (cohort 1) (Supplementary Fig. 1). Our study revealed low (fold change below 0.5) levels of *MKP1* in few grade II and III gliomas, whereas 45% of glioblastomas (20 of 44) displayed low *MKP1* (Fig. 1b, c). The expression of *MKP1* in biopsies obtained from patients with GBM recurrence was also very low (Fig. 1b). Moreover, we determined *MKP1* expression on an independent set of samples from Donostia Hospital (cohort 2), finding similar frequency (14 of 25, 56%) of glioblastomas presenting low expression of *MKP1* (Fig. 1c). When we correlated *MKP1* expression to clinical information, we found that high levels were associated with increased glioblastoma patient overall survival in both cohorts (log rank *p* = 0.04 and 0.007, respectively) (Fig. 1d, e). Indeed, median overall survival of patients with high levels of *MKP1* was 14 and 18 months in cohort 1 and 2, compared to 9 and 6 with low *MKP1*, respectively. Together, these results show that *MKP1* mRNA expression is generally low in tumor samples, lower levels are associated with advanced glioma grade, but these low levels of *MKP1* correlate with poor overall patient survival underlining the impact of MKP1 expression as an independent glioblastoma prognostic marker.

### High levels of MKP1 correlate with GSC differentiation

**Fig. 2:**
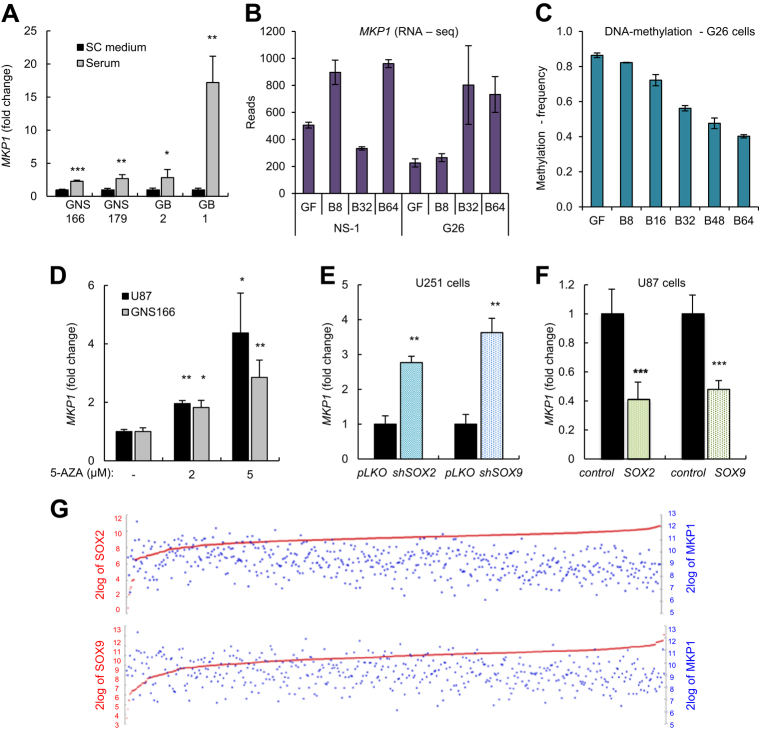
**High levels of MKP1 correlate with GSCs differentiation. a** Analysis of the *MKP1* mRNA expression levels in GSC lines grown in stem medium compared to differentiation conditions (*n* = 3). **b** *MKP1* mRNA expression by RNA-seq analysis in G26 and NS-1 cells at 8, 32, and 64-day time course of BMP treatment after GF withdrawal (GF). Error bars denote SD of four independent experiments. **c** Frequency of *MKP1* promoter methylation after BMP4 continous treatment at the indicated time points. **d** mRNA expression of *MKP1* was analyzed in U87 and GNS166 lines under increasing 5-AZA concentrations. **e** Analysis of *MKP1* mRNA expression levels in U251 cells with knockdown of *SOX2* (*shSOX2*) or *SOX9* (*shSOX9*) related to the control (*pLKO*) condition (*n* = 3). qRT-PCR data are normalized to *GAPDH* expression. **f** *MKP1* levels in U87 cells with *SOX2* or *SOX9* overexpression compared to control cells (*n* = 3). **g** Association analysis of *MKP1* with *SOX2* and *SOX9* in the human glioblastoma TCGA cohort from R2: Genomics Analysis and Visualization Platform http://r2.amc.nl. The analysis showed a negative correlation between *MKP1* and *SOX2* (*R* −0.207, *p* value 1.3e−06), and *MKP1* and *SOX9* (*R* −0.169, *p* value 1.7e−04)

To investigate the association of MKP1 to cellular heterogeneity, we checked its expression in several differentiation conditions, starting with the addition of 1% serum. Of note, *MKP1* levels increased significantly in the four studied GSC lines in a range from 2.5- to 15-fold change after 7 days in the presence of serum (Fig. 2a). GSC exposure to BMP4 induces differentiation both in vitro and in vivo^22^. Therefore, we assessed the expression of *MKP1* in GSCs cultured in the presence of BMP4^23^. Interestingly, RNA sequencing identified *MKP1* within the genes upregulated upon BMP4 induced-differentiation in a GSC line (G26) but also in neural stem cell controls (NS-1) (Fig. 2b). Indeed, the kinetics of transcriptional *MKP1* expression shows an increase from 225 reads after growth factor withdrawal from culture media to 802 and 732 following 32 and 64 days of continuous BMP treatment in G26 cells (Fig. 2b). Moreover, DNA methylation assay revealed that the *MKP1* promoter is demethylated at CpG islands during BMP4 treatment in a time course-dependent manner in G26 cells (Fig. 2c) These results suggest that increased expression of *MKP1* during differentiation is dependent on DNA demethylation of the promoter. To further characterize this idea, we treated both conventional glioma lines and GSCs with the DNA methylation inhibitor 5-AZA. Our results revealed marked induction in *MKP1* levels with increasing concentrations of 5-AZA in U87 and GNS166 cells (Fig. 2d).

Since we observed an inverse correlation between *MKP1* expression and GSC activity in the presence of serum and BMP4, we further studied the association between MKP1 and GSCs, checking the expression of *MKP1* in cells with genetic knockdown of *SOX2* and *SOX9* stem cell factors, which displayed impaired self-renewal^24^. *MKP1* was elevated by >2.5-fold in cells with knockdown of SOX2 or SOX9 (Fig. 2e). On the contrary, *MKP1* levels were reduced in glioma cells with elevated SOX2 or SOX9 activity (Fig. 2f). In line with this evidence, association analysis in the human glioblastoma cohort from the TCGA showed an inverse correlation between *MKP1* and *SOX2* (*r* = −0.207, *p* value = 1.3e−06) and also with *SOX9* (*r* = −0.169, *p* value = 1.7e−04) (Fig. 2g). These results further demonstrate the positive correlation between MKP1 and differentiation of GSCs and reveal that the expression of *MKP1* in GSCs is affected by epigenetic changes and also transcriptionally through, at least, SOX2 and SOX9 transcription factors. This latter effect seems to be specific for GSCs as there is no correlation (negative or positive) between *MKP1*, *SOX2*, and *SOX9* expression in healthy brain tissue (Supplementary Fig. 2).

### MKP1 overexpression inhibits glioma cell tumorigenicity

**Fig. 3:**
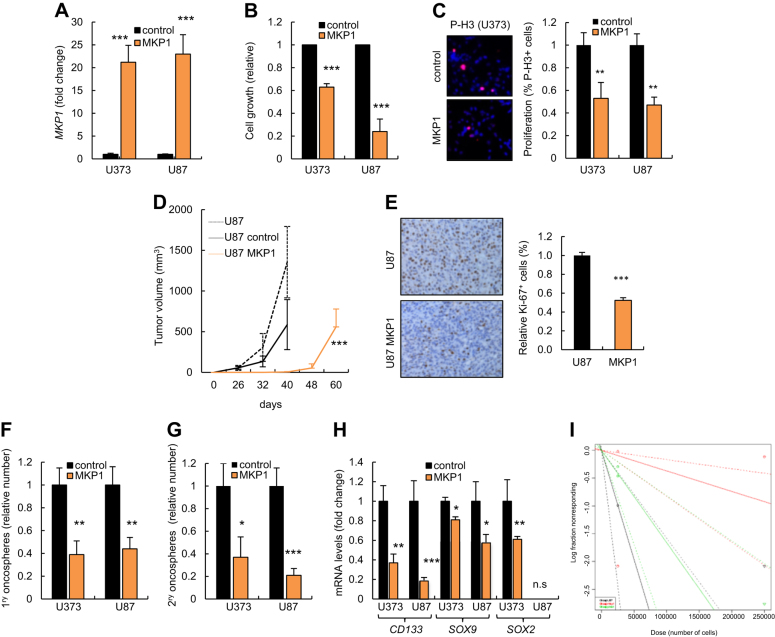
**MKP1 overexpression inhibits glioma cell tumorigenicity. a** mRNA levels of *MKP1* in U87 and U373 cells transduced with the indicated conditions (*n* = 3). **b** Cell growth assay comparing control and *MKP1*-overexpressing U87 and U373 cells (*n* = 3). **c** Representative image and quantification of P-H3^+^ cells in control and *MKP1*-overexpressing U373 cells (*n* = 3). **d** 2.5×10^5^ non-transduced, empty vector (control) and *MKP1*-transduced U87 cells were injected subcutaneously in nude mice (*n* = 8 per condition) and volume of the tumors was scored at the indicated time points. **e** Representative images and quantification of the immunohistochemical staining of Ki67 in tumors from **d** (*n* = 3). **f** Quantification of 1^ry^ oncospheres forming capacity in *MKP1*-infected U373 and U87 cells after 10 days in culture. The numbers are relative to control cells (*n* = 3). **g** Quantification of 2^ry^ oncospheres derived from *MKP1* U373 and U87 cells. The numbers are relative to control cells (*n* = 3). **h** *CD133*, *SOX9*, and *SOX2* expression levels in U373 and U87 cells infected with indicated conditions (*n* = 3). **i** Frequency of tumor initiation after subcutaneous injection in nude mice of 5×10^5^ and 5×10^4^ U87 parental cells or transduced with empty vector, or *MKP1* (*n* = 12). The incidence of tumor initiation was measured using the ELDA platform

Given that clinically high expression of MKP1 positively correlates with patient survival, we overexpressed MKP1 in glioma cell lines expressing low endogenous levels to test the biological consequences of elevating *MKP1* levels with respect to different processes associated with cancer. For this, U87 and U373 cells were transduced with a plasmid encoding *MKP1* and stable *MKP1* overexpression established in these cells (Fig. 3a). First, we observed that high levels of *MKP1* significantly reduced cellular growth (Fig. 3b). Moreover, we analyzed proliferative ability finding that *MKP1* cells exhibited lower rate of proliferation, measured by phospho-Histone3 (P-H3) staining, compared to control cells (Fig. 3c). In order to further investigate whether *MKP1* might regulate tumor cell proliferation and activity, U87 parental, empty vector, or *MKP1*-transduced cells were injected subcutaneously in immunodeficient mice. Of note, there was a delay in tumor formation and a significant decrease in tumor growth in *MKP1*-overexpressing cells compared to controls or U87 parental cells (Fig. 3d). Moreover, the impaired tumorigenic ability of *MKP1* cells was corroborated by immunohistochemistry analysis in the tumors in vivo. Indeed, *MKP1*-derived xenografts possessed lower numbers of Ki67-positive cells than tumors derived from control cells (Fig. 3e). These data reveal that elevated expression of MKP1 suppresses glioma cell tumorigenicity.

U87 and U373 cell lines present a subpopulation of cells with stem cell characteristics that grow as oncospheres^24^. We therefore cultured control and *MKP1*-elevated cells in serum-free GSC media and examined their oncosphere formation ability. In this context, *MKP1* overexpression resulted in a significant reduction of the efficiency of oncosphere formation (Fig. 3f). Moreover, self-renewal activity, measured as the number of secondary oncospheres was also dramatically diminished in cells with elevated *MKP1* levels (Fig. 3g). Consistent with these results, the relative expression of GSC markers such as *CD133*, *SOX2*, and *SOX9* were decreased in oncospheres derived from cells overexpressing *MKP1* (Fig. 3h). These results indicate that MKP1 may suppress the self-renewal activity of GSCs. Tumor-initiating ability through limited dilution injections in immunodeficient mice characterizes functionally the self-renewing population of GSC in vivo^25^. Interestingly, 100% animals injected with 2.5×10^5^ parental or empty vector cells developed tumors 40 days later, while only 12% of *MKP1*-elevated cells presented tumors. Similarly, 50% of mice harboring 2.5×10^4^ empty vector cells generated tumors compared to 0% with elevated *MKP1* (Supplementary Fig. 3). Thus, *MKP1* overexpression exhibited a significant decrease (*p* = 0.001) in the frequency of tumor-initiating cells going from 1/60.235 control cells compared to 1/270.900 *MKP1*-overexpressing cells (Fig. 3i). These results indicate that MKP1 suppresses glioma cell tumor activity not only by inhibiting their proliferative capacity but also decreasing their self-renewal and tumor initiation activity.

### Elevated MKP1 suppresses proliferation and self-renewal ability of GSCs

**Fig. 4:**
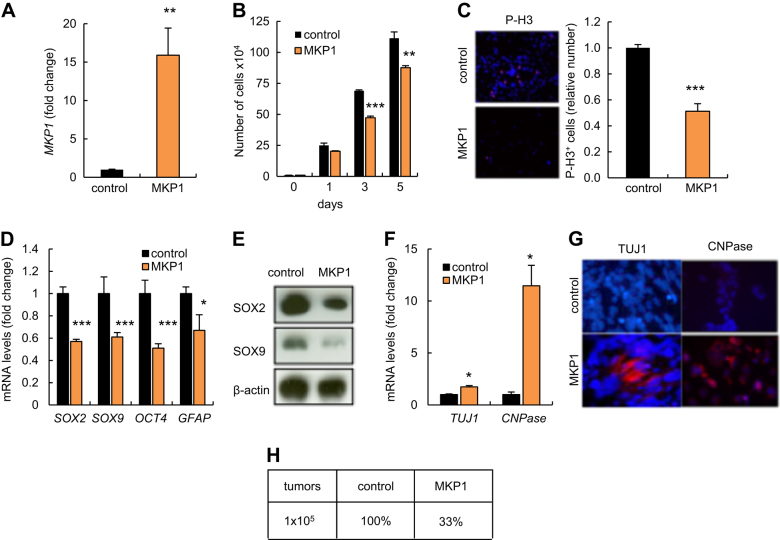
**MKP1 suppresses proliferation and self-renewal ability of GSCs. a** mRNA levels of *MKP1* in GNS166 cells infected with the indicated conditions (*n* = 3). **b** Cell growth assay comparing control and *MKP1*-overexpressing GSCs (*n* = 3). **c** Representative image and quantification of P-H3^+^ cells in both control and *MKP1*-overexpressing GNS166 cells (*n* = 3). **d** *SOX2, SOX9, OCT4*, and *GFAP* mRNA expression levels in GNS166 with ectopic activation of *MKP1* relative to controls (*n* = 3). **e** Representative immunoblots of SOX2 and SOX9 derived from two different and independent retroviral infections. **f** Expression of *TUJ1* and *CNPase* in GNS166 with ectopic *MKP1* relative to control transduced cells (*n* = 3). **g** Representative images of TUJ1^+^- and CNPase^+^-infected GNS166 cells (*n* = 3) after 7 days cultured under differentiation conditions. **h** Control and *MKP1* GNS166 cells were injected stereotaxically in nude mice (*n* = 5 and 3) and growth of the tumors was scored 120 days after

To directly address the impact of MKP1 overexpression in the regulation of GSCs, we stably overexpressed *MKP1* in GNS166 cells. Quantitative RT-PCR confirmed effective overexpression (Fig. 4a); therefore we characterized in detail their proliferative and stem-like cell characteristics. Functionally, high levels of *MKP1* led to a statistically significant decrease in cell proliferation looking at both cell numbers (Fig. 4b) and P-H3 labeling (Fig. 4c), confirming the relevance of MKP1 in glioma cell proliferation. To test the impact of MKP1 in GSC self-renewal and differentiation, we studied the relative expression of several stem cell marker genes; *SOX2*, *SOX9*, *OCT4*, and *GFAP* finding that all of them were decreased in *MKP1*-overexpressing cells by qRT-PCR (Fig. 4d), and SOX2 and SOX9 by western blotting (Fig. 4e). On the contrary, differentiation markers such as *TUJ1* and *CNPase* were upregulated at a transcriptional level (Fig. 4f), a result further substantiated by immunofluorescence analysis in which we found higher number of TUJ1 and CNPase-positive cells in GNS166 overexpressing *MKP1* compared to control cells (Fig. 4g). Finally, we assessed whether MKP1-elevated cells remained tumorigenic. For this, cells were injected into the ventral forebrain of immunocompromised mice and we then monitored for symptoms of glioma development. While 100% of animals injected with control cells became sick and developed tumors at 4 months post injection, only 33% of animals injected with cells overexpressing *MKP1* developed tumors (Fig. 4h). In summary, these results demonstrate that genetic overexpression of *MKP1* results in decreased self-renewal and tumorigenic potential. Overall, these data also raise the prospect that the induction of MKP1 could be exploited for therapy in glioblastoma.

### Activation of JNK and p38 MAPK is impaired by MKP1 in glioma cells

**Fig. 5:**
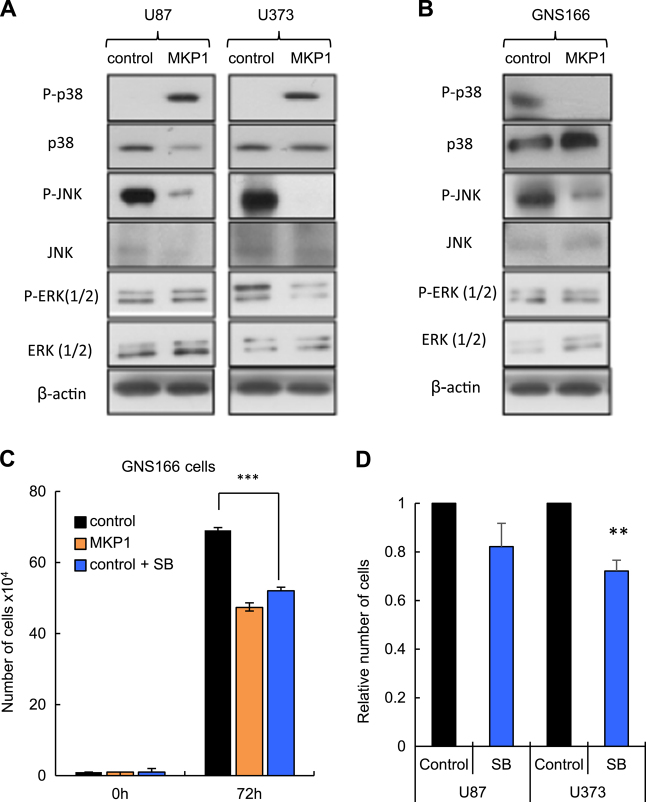
**Activation of JNK and p38 MAPK is impaired by MKP1 in glioma cells. a**,** b** Representative western blot of the effect of overexpressing *MKP1* in MAPK activity in (**a**) U87, U373 and (**b**) GNS166 cells. **c** Cell growth assay comparing control and *MKP1*-overexpressing GNS166 cells after 72 h of treatment with p38 MAPK inhibitor SB203580 (*n* = 3). **d** Cell growth assay of U87 cells comparing control and SB203580-treated cells for 72 h (*n* = 3)

MKP1 is a negative inhibitor of the activity of JNK, p38 MAPK, and ERK1/2. In an effort to identify the downstream molecular pathways of MKP1 activity on glioma cells, we tested the total and phosphorylated expression of these proteins in cells with elevated *MKP1*. We did not see marked differences in the total JNK, p38 MAPK, or ERK expression in glioma cells (U87, U373) or in GNS166 cells (Fig. 5a, b). However, there were notable differences with respect to their activated forms in these cellular contexts. First, we found that P-ERK was not altered in any of the cell lines (Fig. 5a, b) and only slightly in U373 cells, suggesting that MKP1 function on glioblastoma is not dependent in this kinase. Next, we measured the activation of p38 MAPK, observing contradictory results. As expected, P-p38 MAPK was decreased in GNS166 cells derived from patients (Fig. 5b), but its expression was increased in U87 and U373 conventional cells (Fig. 5a). We tried to explore further this opposite activity and cultured the different cells lines with SB203580, an inhibitor of p38 MAPK activity observing that treatment with this compound significantly decreased GSCs proliferation by 35% and to a level observed in cells overexpressing *MKP1* (Fig. 5c), whereas the effect in proliferation was not statistically significant and so clear in U87 (18%) and U373 (25%) cells (Fig. 5d). Finally, we measured P-JNK activity and found that high levels of MKP1 reduced significantly its levels both in U87 and U373 glioma cells and GNS166 cells (Fig. 5a, b). These results suggest that JNK might be the main effector of MKP1 activity in glioma cells.

### High levels of MKP1 correlate with temozolomide sensitivity

**Fig. 6:**
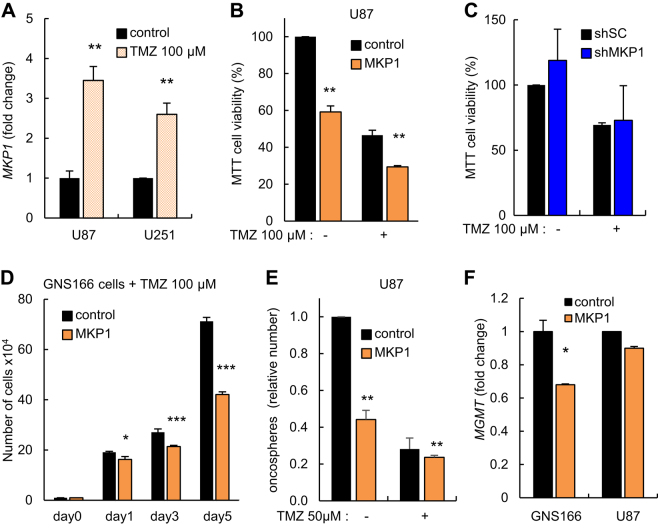
**High levels of MKP1 sensitize glioma cells to temozolomide treatment. a** mRNA expression of *MKP1* was studied in U87 and U251 GBM cell lines under the effect of TMZ (100 μM) for 24 h. **b** Cell viability comparing control and *MKP1*-overexpressing U87 cells under 100 μM TMZ treatment for 72 h. **c** Cell viability comparing U87 cells transfected with shSC or sh*MKP1* cultured with 100 μM TMZ for 72 h. **d** Cell proliferation comparing control and *MKP1*-overexpressing GNS166 cells under 100 μM TMZ treatment. **e** Relative oncosphere-forming capacity of indicated U87 cells in presence of 50 μM TMZ for 7 days. **f** *MGMT* mRNA levels were assayed in both control and *MKP1*-transduced GNS166 and U87 cells. Statistical significance was obtained with Student’s *t* test (*p ≤ 0.05; **p ≤ 0.01; ***p ≤ 0.001)

Glioblastomas are known to be resilient to therapy treatment because malignant GSCs survive radiotherapy and chemotherapy with TMZ alkylating agent first-line treatment. We studied the impact of MKP1 in response to TMZ treatment. First, we found that short-term exposure to 100 µM of TMZ induced the expression of *MKP1* in U87 and U251 glioma cell lines by more than threefold (Fig. 6a), suggesting that MKP1 levels could be involved in response to current chemotherapy. To determine this idea, U87 cells with ectopic elevation of *MKP1* were exposed to the same concentration of TMZ for 72 h and cell chemosensitivity was measured by MTT assay. Notably, U87 cells with high levels of *MKP1* presented increased sensitivity (% of viability <30%) when compared to control cells (% of viability of 50%) in the presence of TMZ (Fig. 6b). On the contrary, TMZ did not induce any significant effect in U87 cells transduced with a *shMKP1* (Fig. 6c), likely because the endogenous *MKP1* levels were already very low (Fig. 1a, Supplementary Fig. 4).

In order to further test the involvement of *MKP1* in temozolamide (TMZ) response, we examined the sensitivity of GSCs with high levels of MKP1 to chemotherapy. We measured the proliferative capacity of GNS166 cells in the presence of TMZ finding that cells with elevated *MKP1* showed significant drug sensitivity and impaired cell growth when compared to control cells (Fig. 6d). In line with these results, the formation of oncospheres was also significantly reduced in U87 cells with elevated *MKP1*, when treated with TMZ (Fig. 6e). These findings confirm that high levels of MKP1 increase chemosensitization to TMZ. The presence of the DNA-repair protein MGMT leads to cellular resistance to cytotoxic actions of TMZ, whereas methylation of the *MGMT* promoter increases TMZ cellular sensitivity and increases patient survival^2, 26^ Therefore, we tested whether there was a correlation between high levels of *MKP1* and *MGMT* promoter status in human biopsies in vivo. The percentage of *MGMT* methylated cases in biopsies with *MKP1* high and low expression did not show statistically significant differences in a small cohort of 42 patients (data not shown), however, *MGMT* expression was decreased in U87 and GNS166 cells stably overexpressing *MKP1* compared to control cells (Fig. 6f). These results indicate that high levels of *MKP1* may function as a TMZ sensibilizer in human GSCs.

### HDAC inhibitors upregulate MKP1 in glioma cells

**Fig. 7:**
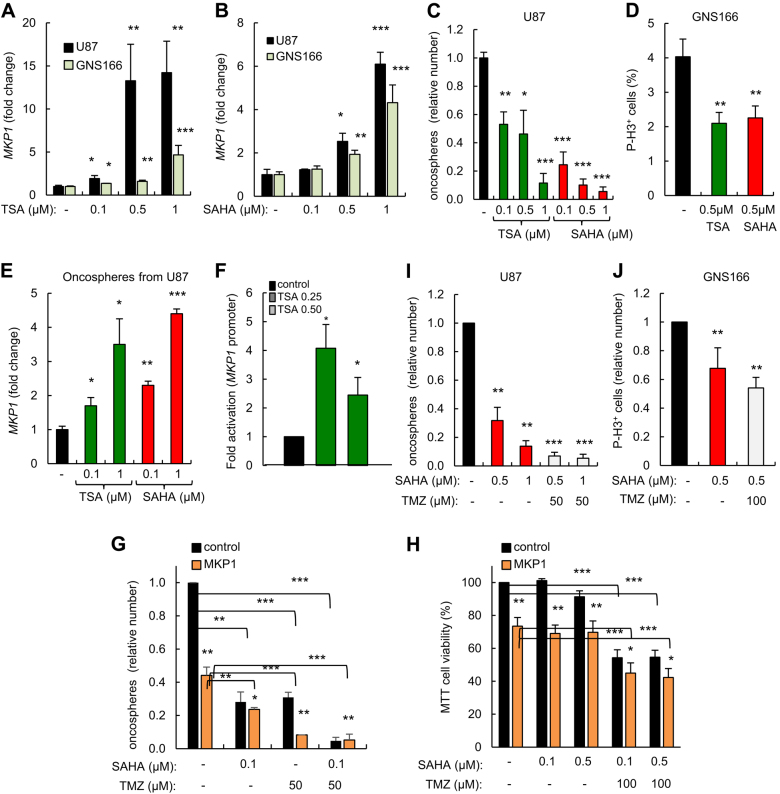
**HDAC inhibitors upregulate MKP1 in glioma cells and sensitizes to temozolomide treatment. a** *MKP1* mRNA levels were studied in U87 and GNS166 cell lines under 24 h of treatment with increasing doses of TSA (0.1, 0.5, and 1 µM) and **b** SAHA (0.1, 0.5, and 1 µM). *MKP1* levels were assayed by qRT-PCR data, are normalized to *GAPDH* expression, and are relative to non-treated cells. **c** Quantification of oncospheres forming capacity in TSA- and SAHA-treated U87 cells after 7 days in culture. The numbers are relative to control cells (*n* = 3). **d** Quantification of P-H3^+^ cells in TSA- and SAHA GNS166-treated cells (*n* = 3). **e** Expression of *MKP1* in U87 derived oncospheres in the presence of indicated concentrations of HDAC inhibitors. Levels are relative to non-treated cells (*n* = 3). **f** U87 cells were co-transfected with *pGL3-MKP1* luciferase promoter construct and increasing doses of TSA (control (0), 0.25, and 0.5 µM) for 48 H. Levels are relative to control condition (*n* = 3). **g** Oncosphere formation after 7 days with 0.1 and 0.5 μM SAHA, 50 μM TMZ, or the combination of both compounds. **h** Cell viability comparing control and *MKP1*-overexpressing U87 cells cultured with 0.1 and 0.5 μM of SAHA alone or with 100 μM TMZ treatment for 72 h. The numbers are relative to control, non-treated cells. **i** Quantification of oncospheres formed after 7-day treatment with SAHA (0.5 µM) and combined SAHA+TMZ (50 µM) in U87 cells. The numbers are relative to control, non-treated cells (*n* = 3). **j** Quantification of P-H3^+^ cells in untreated, SAHA (0.5 µM), and combined SAHA (0.5 µM) +TMZ (100 µM)-treated GNS166 cells (*n* = 3)

Epigenetic alterations are increasingly implicated in glioblastoma pathogenesis and we have observed that MKP1 levels are epigenetically modified in GSCs. Histone deacetylase inhibitors (HDACIs) are a group of promising epigenetic agents for glioblastoma^27^, which have been shown to upregulate the expression of *MKP1* in lung cancer^28^. Therefore, we tested the effect of two epigenetic agents, trichostatin A (TSA), and suberoylanilide hydroxamic acid (SAHA), on MKP1 expression. We observed a remarkable increase in *MKP1* with augmented doses of both HDACIs in U87 and in GNS166 cells (Fig. 7a, b). Importantly, this decline correlated with marked dose-dependent reduction in oncosphere formation (over 60%) in U87 (Fig. 7c), and decrease in the proliferative capacity (by 50%) of GNS166 cultured with increasing concentrations of TSA and SAHA (Fig. 7d). Of note, the expression of *MKP1* on the oncospheres generated in U87 cells in the presence of both HDACIs also displayed significantly higher levels of *MKP1* compared to non-treated controls (Fig. 7e). These findings indicate that HDACIs can inhibit both proliferative ability and oncosphere formation potential of glioma cells concomitantly with elevation of MKP1 expression. We further study the effect of the HDACIs on MKP1 activity, and found that increasing concentrations of TSA elevated the luciferase activity of the *MKP1* promoter (Fig. 7f). Moreover, U87 cells ectopically overexpressing *MKP1* generated fewer oncospheres than control cells when treated with 0.1 μM of SAHA (Fig. 7g). They also exhibited decreased cell viability as measured by MTT assays (Fig. 7h). These findings suggest that MKP1 represents a candidate biomarker for HDAC inhibitors sensitivity in glioblastoma.

Combined therapeutic approaches acting synergistically have been proven more effective than individual treatments. We therefore tested whether HDACis could represent a potential enhancer of the cytotoxic effects of TMZ and sensitize cells with different levels of *MKP1*. Accordingly, we performed oncosphere formation assays in which U87 cells were cultured with a constant dose of 100 μM TMZ together with 0.5 and 1 μM of SAHA (concentrations that significantly elevated *MKP1* expression). Interestingly, combined treatment of SAHA and TMZ resulted in a higher decline in oncosphere formation than with the single agent alone (Fig. 7i). Moreover, we cultured GNS166 cells with this combined therapeutical strategy finding that there was also a synergistic anti-proliferative effect of SAHA and TMZ in those cells (Fig. 7j). Finally, to evaluate whether the anti-tumoral activity of the combination of SAHA and TMZ might be directed thorough GSCs and mediated by *MKP1*, we repeated the oncosphere formation and MTT assays in control and *MKP1*-overexpressing U87 cells cultured with SAHA and TMZ in combination. Notably, combined treatment of SAHA and TMZ formed significantly lower number of oncospheres and reduced cell viability from *MKP1*-overexpressing cells than with single agents alone or from control cells (Fig. 7g, h). Overall, our results demonstrate that combined treatment of HDACis and TMZ achieved a strong anti-tumoral effect and identify *MKP1* as a novel biomarker for this therapeutic strategy.

## Discussion

In this study, we have identified that MKP1/DUSP1 expression is heterogeneous within low- and high-grade gliomas and particularly in glioblastoma samples. Importantly, we have demonstrated that the subset of biopsies overexpressing *MKP1* levels is associated with the subgroup of patients with elevated median overall survival. Remarkably, this expression and clinical relevance is differential in relation to additional types of cancer with opposite expression. Indeed, the role of MKP1 in cancer is mostly correlated with carcinogenesis, high expression levels of MKP1 promoting tumorigenesis in prostate, pancreatic, colon, bladder, gastric, breast, and lung cancer^20, 21, 29–33^. Moreover, MKP1 has also been positively related to tumor progression in lung cancer (by downregulating JNK and p38 kinases)^21, 34^, in breast cancer by inducing epithelial mesenchymal transition and PKC pathway, and in hepatocellular carcinoma by inhibiting ERK activity^35^. On the other hand, a few studies have reported a negative role of MKP1 in carcinogenesis, such as in hepatocellular carcinoma and head and neck cancer^35–38^, similar to the situation we find with glioblastomas.

TMZ is currently the most effective chemotherapy for glioblastoma. Indeed, its incorporation in the clinic extended patient median survival from ~12 to 15 months^2, 39^. Damage generated by TMZ can be repaired by MGMT, thus inducing treatment resistance, while methylation of the MGMT promoter leads to an increase in TMZ sensitivity^26^. Our results show that *MKP1* is overexpressed in response to TMZ and cells with elevated levels of *MKP1* express lower levels of MGMT. Moreover, functional studies in cells ectopically overexpressing or silencing *MKP1* confirm that cells with elevated levels of *MKP1* are more sensitive to TMZ explaining the correlation observed between high levels of *MKP1* and elevated patient survival in clinical biopsies. These results also support the fact that *MKP1* activity is context-dependent, because its high levels induce therapy resistance in other types of cancers such as lung (to *cis*platin), ovary (*cis*platin)^20^, pancreatic (gemcitabine)^40^, breast (taxanes)^41^, and U937 cells (radiation)^42^. The resistance against different therapies is related in most of the tumors to the downregulating activity of MAP kinases, JNK, and p38 MAPK, which are required for induction of apoptosis after those treatments^20^. In glioma cells, MKP1 activity appears to be related to GSC activity because we have observed a positive correlation between GSC differentiation and its elevated levels.

In addition to an association between higher levels of *MKP1* expression in biopsies with overall higher survival rates of patients with glioblastomas, we showed that genetic overexpression of *MKP1* in glioma cells and directly in the GSC population decreases proliferative ability, causes depletion of self-renewal, and subsequently decreases tumor initiation and progression. We also identified that *MKP1* modulates glioma cell heterogeneity. In agreement with this, we observed enrichment of *MKP1* in differentiated GSC upon treatment with BMP4 and serum addition, induction of GSC differentiation in MKP1-overexpressing cells and modulation of its expression in cells with altered activity of SOX2 and SOX9, well-established stem cell genes and regulators of GSC activity^43^. Our results indicate that the activity of MKP1 is negatively involved in GSC maintenance likely regulating the interplay between proliferation, self-renewal, and differentiation. These activities are likely to be mediated by SOX factors and JNK kinase. Indeed, we identified that there is an inverse correlation between *MKP1* and *SOX9* and *SOX2* expression in glioblastoma samples. Moreover, we have found that high levels of MKP1 in glioma cells and also GSCs, downregulate JNK phosphorylation. Notably, increased JNK phosphorylation is related with the tumor-initiating capacity of GSC and carcinogenesis^44^. Along the same lines, activation of the c-jun/MELK pathway has been reported to maintain GSCs in immature stage and facilitate radiotherapy resistance^45^. In addition to transcriptional regulation via SOX2 and SOX9 stem cell factors, the activity of MKP1 is also affected by epigenetic changes. Indeed, we have observed that the MKP1 promoter is demethylated upon BMP4 differentiation treatment. Similarly, pharmacological demethylation with 5-aza also increases the expression of *MKP1*. These findings are supported with the evidence that, during carcinogenesis, the epigenome of cancer cells undergoes multiple alterations that facilitate cell plasticity and are relevant for cancer progression^46^.

**Fig. 8:**
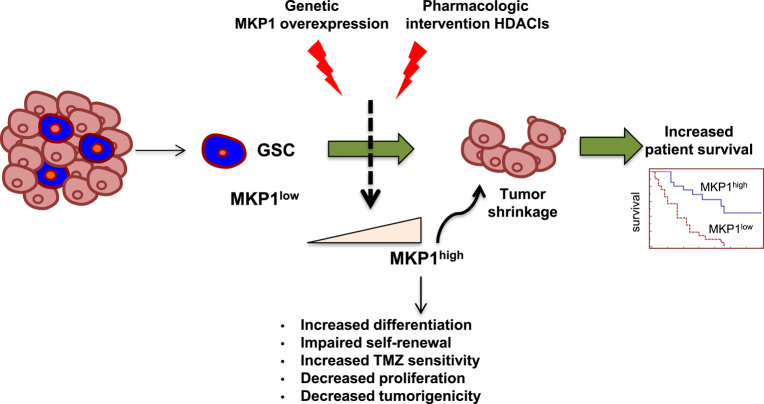
**Schematic image representing the function of MKP1 on glioma stem cell (GSC) activity and glioblastoma progression.** Glioblastoma is a heterogeneous tumor with a population of GSCs (in blue, chemo-, and radiotherapy-resistant) with low levels of MKP1. Genetic MKP1 overexpression or its upregulation with HDACIs increases differentiation, impairs tumor growth, and sensitizes cell to TMZ indicating that elevation of MKP1 might be and interesting approach to extend patient survival and avoid tumor recurrence

Since elevated levels of *MKP1* have been associated with overall extended survival of patients, TMZ chemosensitivity, and GSC differentiation, our results suggest that targeting the activity of MKP1 (through induction) may offer a new promising therapeutic treatment modality in glioblastoma (Fig. 8). Previous investigators have observed that MKP1 levels are elevated with Notch inhibitors (γ secretase) and HDACIs^28, 47^. In an effort to identify drugs or molecules that might activate efficiently the expression of MKP1, we tested the latter using two different compounds (TSA and SAHA). Notably, we observed that MKP1 expression is regulated by epigenetic modifications in glioma cells and, HDACIs are a group of epigenetic molecules with promising results in pre-clinical and clinical trials in gliomas and in particular with glioblastomas^27, 48^, and some (SAHA, romidepsin and bellinostat) have received approval from the FDA for the treatment of cutaneous T-cell lymphoma. We found that TSA and SAHA increased significantly, between 2 and 15-fold, the expression of *MKP1*. The elevated expression level detected by qRT-PCR may be due to an increased activity of the *MKP1* promoter. The increase was dose-dependent and already apparent at low doses. Moreover, the citotoxic activity of HDACIs is higher in cells with elevated *MKP1* expression postulating it as a biomarker for these compounds. These results are particularly important in the case of SAHA, which is being tested in clinical trials for glioblastoma alone or in combination with current therapy treatments, such as TMZ or radiotherapy^27^. Our results demonstrate that the pharmacological overexpression of MKP1 may be feasible using inhibitors of HDACs and could be relevant for these trials.

It is important to note that our results and previous investigations demonstrate that these agents impair self-renewal activity, induces differentiation, and subsequently decreases tumorigenic activity^49, 50^. Given that increased MKP1 expression also induces sensitivity to TMZ, a combined treatment of HDACIs and TMZ might result in improved response to therapy in gliomas. Indeed, we observed a synergestic anti-tumor effect of the combination of TMZ and SAHA, which is more prominent as higher are the cellular levels of MKP1. These results support previous glioblastoma pre-clinical studies^51^ and suggest that MKP1 represents a candidate biomarker for this therapeutic strategy in glioblastoma. Overall, our data identifies MKP1 as a central player in glioblastoma, since it modulates GSC activity. In fact it has a dual role as it acts as an inhibitor of proliferation and promoter of differentiation (Fig. 8). These data provide further evidence for the development of HDACIs as potential therapeutics against this type of cancer, particularly in combination with TMZ and using MKP1 as a biomarker and a strong rationale for the development of novel strategies targeting MKP1 for glioblastoma.

## Materials and methods

### Patients and tumor samples

Human glioblastoma samples and patient clinical information were obtained from Hospital Valencia (cohort 1) and Donostia University Hospital (cohort 2). The study included biopsies from 44 patients seen in Valencia and 25 in San Sebastian, and diagnosed as primary glioblastoma grade IV according to the WHO criteria. Basque Biobank for Research O+EHUN http://www.biobancovasco.org provided the cohort 2. All study participants signed informed consent forms approved by the Institutional Ethical Committee. The study was approved by the ethic committee of Biodonostia Institute and Hospital Donostia.

### Cell lines, cultures, and reagents

Glioma cell lines U87MG (U87), U373MG (U373), U251MG (U251), A172, and T98G were purchased from the ATCC. The cell lines were cultured in DMEM (Gibco), supplemented with 10% FBS (Gibco), 100 U/ml penicillin, and 100 µg/ml streptomycin for traditional monolayer cultures or in in GSC medium consisting of DMEM/F-12 (Sigma) supplemented with N2, B27 supplements (Fisher) and growth factors (20 ng/ml basic fibroblast growth factor (bFGF), and 20 ng/ml epidermal growth factor (EGF)) (Sigma) for oncosphere cultures, similarly to glioblastoma primary tumors. Cells were maintained at standard conditions of 37 °C, 5% CO_2_ in humidified atmosphere, were dissociated, and cells grown in oncosphere medium for 10 days. Differentiation assays were performed by adding 1% FBS to the DMEM-F12 complete medium. Temozolomide (TMZ) (Sigma), SB203580 (Selleckchem), SAHA (Cayman), TSA (Sigma), and 5-Aza-2′-deoxycytidine (5-AZA) (Sigma) were dissolved in DMSO.

### Oncosphere assays

U87 and U373 were grown in GSCs medium for 10 days. These oncospheres were then disaggregated with accutase (Gibco), and seeded for secondary (2^ry^) oncospheres and maintained for another 10 days. For quantification studies, 500 cells per well were seeded in non-treated 12-wells flat bottom plates and fresh media was added every 3 days to the plate. After 10 days, oncospheres were counted. Same procedure was performed for 2^ry^ oncosphere assay.

### Lentiviral infections and Luciferase assays

Infections were performed as previously described^30^. For stable overexpression of *MKP1*, the retroviral plasmid pLXSN MKP1 was used, whereas pGIPZ scrambled (*shSC*) empty vector and *pGIPZ shMKP1* were used for silencing experiments. For stable overexpression of *SOX2* and *SOX9*, *pLM-mCitrine-SOX2* (a gift from Ander Izeta, Biodonostia Institute) and *pWXL SOX9* (a gift from Bob Weinberg, Addgene plasmid 36979) lentiviral constructs were used. For *SOX2* or *SOX9* knockdown, cells were infected with *pLKO.1 shSOX2* (a gift from M. Meyerson, Addgene plasmid 26353), *shSOX9* (a gift from B. Weinberg, Addgene plasmid 1855), or empty vector. Infected cells were selected in the presence of 2 μg/ml puromycin or 1.5 μg/μl of geneticine depending on the selectable marker of the plasmid and then maintained with 0.2 μg/ml puromycin or geneticine (Sigma).

For luciferase assays, U87 cells were transfected with a *MKP1*-promoter luciferase construct (*pGL3 MKP1*) (250 ng), and cultured with increasing concentrations of TSA. The relative luciferase activity was determined after 48 h of transfection using the dual-Luciferase reporter assay system (Promega). Results are average of three independent experiments (each sample is by triplicate in each experiment) and expressed as mean values ± SD).

### RNA analysis

Total RNA was extracted with Trizol (Life Technologies). Reverse transcription was performed using random priming and Superscript Reverse Transcriptase (Life Technologies). Quantitative real-time PCR was performed using Absolute SYBR Green mix (Thermo Scientific) in an ABI PRISM 7300 thermocycler (Applied Biosystems). Variations in input RNA were corrected by subtracting the number of PCR cycles obtained for *GAPDH*.

### Western blot analysis

Immunoblots were performed following standard procedures. The antibodies for detection of SOX2 (AB5603 Millipore), SOX9 (AB5535 Millipore), p-p38 (9211 Cell Signalling), p38 (sc-7972 Santa Cruz), p-JNK (9251 Cell Signalling), JNK (sc-474 Santa Cruz), p-ERK1/2 (9101 Cell Signalling), ERK1/2 (9102 Cell Signalling), and ß-actin (AC-15 Sigma) were used in the study. For secondary antibodies, we used horseradish peroxidase (HRP)-linked anti-rabbit or anti-mouse (SantaCruz Biotechnology), both at a 1:2000 dilution. Detection was performed by chemiluminescence using NOVEX ECL Chemi Substrate (ThermoFisher).

### Immunofluorescence

Cells were fixed with 4% paraformaldehyde for 15 min, and washed with phosphate-buffered saline (PBS) supplemented with 0.3% Triton X-100 and 1% FBS, for 5 min at 4 °C. Subsequent to blocking for 1 h with PBS and 1% FCS, cells were incubated with p-Histone3 (P-H3), (ab14955 Abcam), neuron-specific class III beta-tubulin (TUJ1), (MMS-435P Covance), cyclic nucleotide phosphodiesterase (CNPase), (MAB326 Millipore) antibodies for 2 h. Secondary antibody was Alexa Fluor 555 rabbit anti-mouse IgG. Nuclear DNA was stained with 4′,6-diamidino-2-phenylindole (DAPI) (Sigma).

### Immunohistochemistry

For immunohistochemistry, tumor sections were incubated with primary antibody for Ki67, ab15580, Abcam. Sections then were incubated with MACH 3 Rabbit Probe and MACH 3 Rabbit HRP-Polymer (M3R531, Biocare Medical).

### In vivo carcinogenesis and survival assays

All process involving animals were subjected to approval by the Research Animal Care of Biodonostia Institute. U87 cells were collected with trypsin/EDTA and resuspended in PBS before subcutaneous injection. For tumor initiation, 2.5x10^5^ and 2.5x10^4^ cells were injected subcutaneously into both flanks of Foxn1^nu^/Foxn1^nu^ nude mice (8 weeks old and randomly selected) and frequency of tumor initiation was measured with ELDA. For tumor growth, mice were observed on a weekly basis and external calipers were used to measure tumor size, and from these measurements, tumor volume was estimated by *V* = *L*×*W*^2^×0.5; where *L* is the tumor length and *W* is the tumor width. The investigator was blinded to the group allocator. For xenotransplantation, GNS166 cells were injected stereotactically into the frontal cortex of 6–8-week-old NOD-SCID mice. Briefly, GSCs were disaggregated with accutase (Gibco) and resuspended in PBS. Cells of 1×10^5^ were injected into the putamen using a stereotaxic apparatus. Survival distributions were determined using the log-rank test and GraphPad Prism 5 software.

### Data analysis

Results are represented as mean values ± SEM, indicating the number of experiments carried out for each assay. Statistical significance has been calculated using Student’s t test, (*p ≤ 0.05; **p ≤ 0.01; and ***p ≤ 0.001), or the log-rank test for Kaplan–Meier survival analyses. Additional tests are included in the text.

## Supplementary information

Figure Legends of supplementary results
Supplementary Fig. 1
Supplementary Fig. 2
Supplementary Fig. 3
Supplementary Fig. 4
