# Supplementary material for: High expression of MKP1/DUSP1 counteracts glioma stem cell activity and mediates HDAC inhibitor response
Source: Oncogenesis. 2017 Dec 14;6:401. doi: 10.1038/s41389-017-0003-9 (PMC5865544; doi:10.1038/s41389-017-0003-9)
Supplement: Supplementary file 1 — Figure Legends of supplementary results [file 41389_2017_3_MOESM1_ESM.docx]

**Supplementary figures**

**Figure Legend 1.** Expression of *MKP1* mRNA in each of the samples included in a cohort from de Hospital La Fe from Valencia containing 2 glioma grade II, 13 glioma grade III and 44 glioblastomas. qRT-PCR data are normalized to *GAPDH* expression and expression in tumors is relative to healthy brain tissue included as an average of 6 brain tissues and marked as C (control, 4 line starting from the right). The last 3 squares show low levels of MKP1 in T98G, U373 and U87 glioma cell lines

**Figure Legend 2.** Association analysis of *MKP1* with *SOX2* and *SOX9* in control samples from the human glioblastoma TCGA cohort from ‘R2: Genomics Analysis and Visualization Platform (http://r2.amc.nl).

Figure Legend 3. Frequency of tumor formation after subcutaneous injection of U87 parental cells and transduced with empty vector (control) or MKP1 plasmid in nude mice. The incidence of tumor initiation was measured 40 days post-injection.

Figure Legend 4. U87 cells transfected with pGIPZ scrambled (*shSC*) vector and *pGIPZ shMKP1* and selected with puromycin. Expression of *MKP1* mRNA in U87 stably carrying *shSC* or *shMKP1* 48 hours post-selection. qRT-PCR data are normalized to *GAPDH* expression.
