# Supplementary figures and images for: High expression of MKP1/DUSP1 counteracts glioma stem cell activity and mediates HDAC inhibitor response
Source: Oncogenesis. 2017 Dec 14;6:401. doi: 10.1038/s41389-017-0003-9 (PMC5865544; doi:10.1038/s41389-017-0003-9)

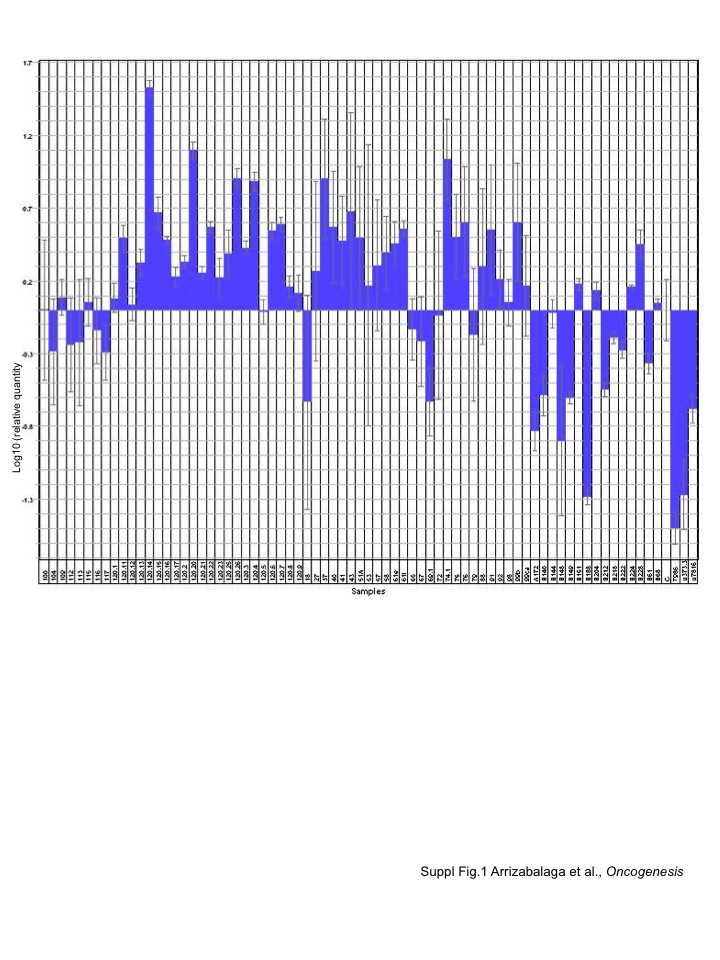

Supplement: Supplementary file 2 — Supplementary Fig. 1 [file 41389_2017_3_MOESM2_ESM.jpg]

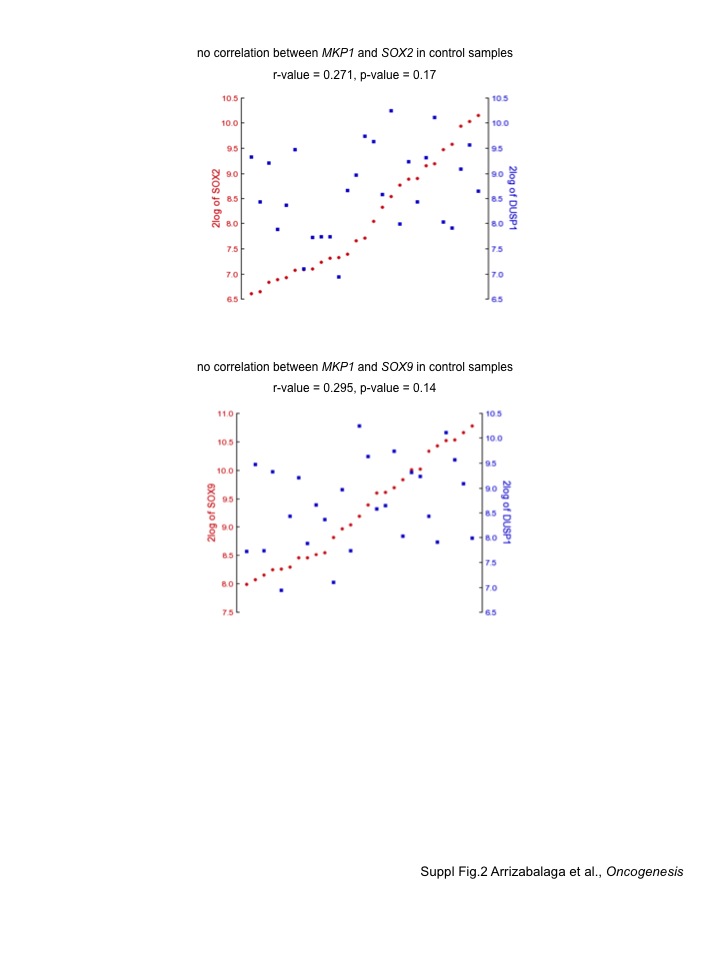

Supplement: Supplementary file 3 — Supplementary Fig. 2 [file 41389_2017_3_MOESM3_ESM.jpg]

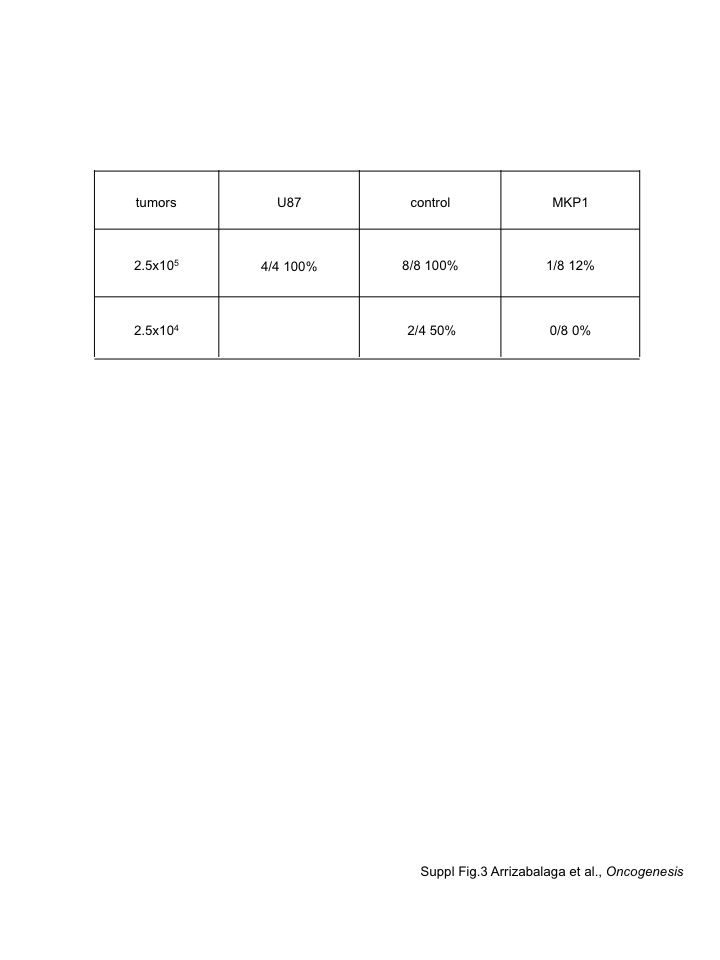

Supplement: Supplementary file 4 — Supplementary Fig. 3 [file 41389_2017_3_MOESM4_ESM.jpg]

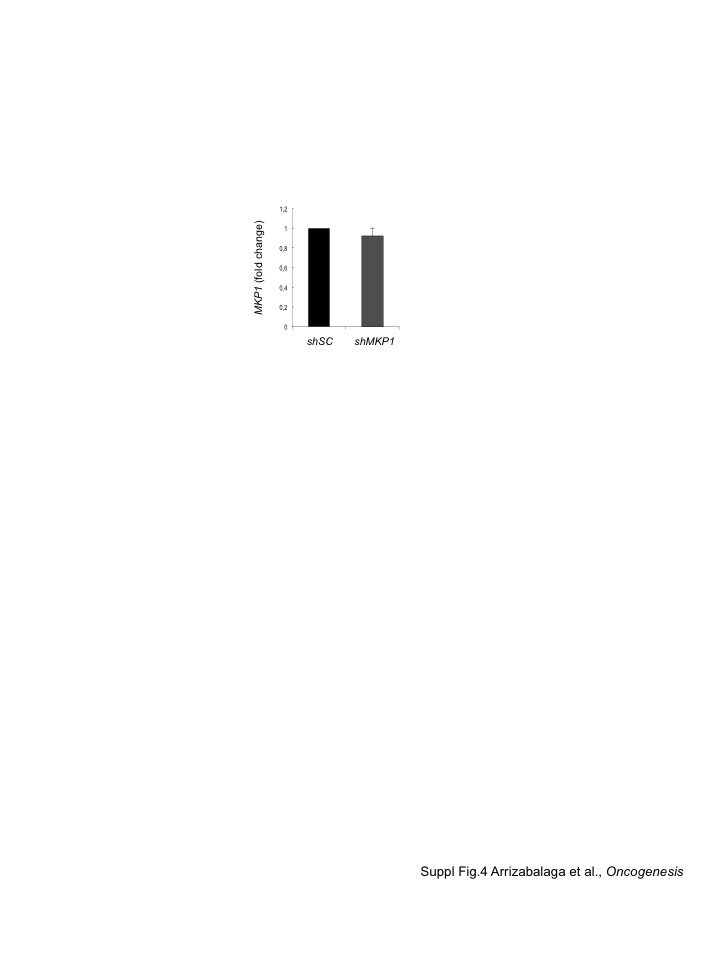

Supplement: Supplementary file 5 — Supplementary Fig. 4 [file 41389_2017_3_MOESM5_ESM.jpg]
